# Right hemicolectomy for ileocolonic intussusception in an adult with active COVID-19 infection: a case report

**DOI:** 10.1093/jscr/rjab205

**Published:** 2021-06-10

**Authors:** Katherine M Jackson, Aaron L Sabbota

**Affiliations:** University of Rochester Medical Center, Department of Surgery, Rochester, NY 14642, USA; University of Rochester Medical Center, Department of Surgery, Rochester, NY 14642, USA

## Abstract

The most common symptoms of Severe Acute Respiratory Syndrome Coronavirus 2 (SARS-CoV-2) are fevers, fatigue and dry cough. However, growing data suggest gastrointestinal (GI) manifestations occur in the majority of patients. Small bowel obstruction remains a significant cause of surgical abdominal emergencies in the adult population, although most cases are secondary to adhesive disease. We present a case of ileocolonic intussusception in an adult with active COVID-19 infection. Our patient presented with small bowel obstruction 4 days after diagnosis of COVID-19 with typical respiratory symptoms. Imaging revealed ileocolonic intussusception and possible cecal mass for which a right hemicolectomy was performed. Recovery was unremarkable. Pathology suggested necrosis without an identifiable mass. To the best of our knowledge, this is the first documented case of small bowel obstruction secondary to ileocolonic intussusception in an adult related to GI manifestation of COVID-19.

## INTRODUCTION

The COVID-19 pandemic has infected over 130 million people across the globe [1]. The most common symptoms include fever, fatigue and a dry cough [2]. Initially thought to be infrequent, recent investigations have shown that up to 50% of patients may experience gastrointestinal (GI) manifestations [3].

Small bowel obstruction (SBO) accounts for up to 16% of surgical admissions, with ~24% requiring surgical intervention [4, 5]. Most cases of SBO in adults is secondary to adhesive disease and hernias, representing 65% and 10% of cases in the USA [6]. In adults, intestinal intussusception, or ‘telescoping’ of the bowel, represents only 1% of intestinal obstructions, but vast majority of patients undergo operative intervention [7, 8].

Worldwide, four pediatric patients with intussusception secondary to COVID-19 infection have been reported [3]. Here, we present the first known case of intestinal obstruction requiring operative intervention in an adult with an active COVID-19 infection.

## CASE REPORT

**Figure 1:**
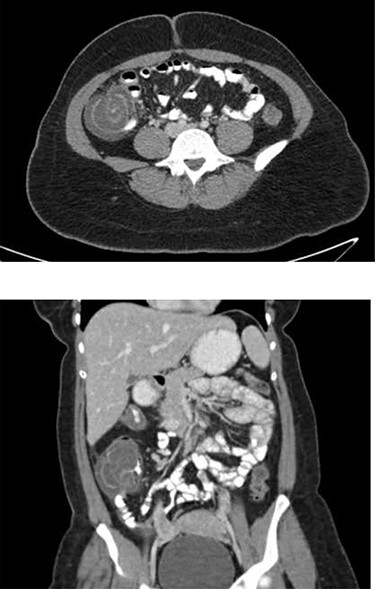
Representative axial (top) and coronal (bottom) images from patient’ s CT abdomen/pelvis with PO and IV contrast demonstrating cecal thickening and ileocolic intussusception.

A 25-year-old female with a history of hypertension and peripartum pericarditis 2 years prior (recent echocardiogram normal) presented to the emergency department (ED) with a sore throat, fevers and myalgias. She had no diarrhea, constipation or blood in stool noted. On exam she had a fever to 38.7°C and tachycardia, and normal oxygen saturations on room air. Complete blood cell count and basic metabolic panel were not performed. She tested positive for COVID-19 infection via nasopharyngeal polymerase chain reaction (PCR), and was discharged home. She presented 4 days later to the ED with 1 day of intermittent severe cramping abdominal pain located in her right lower quadrant. She reported new onset nausea, vomiting and bright red blood in the stool. She was febrile to 38.2°C, heart rate was 92 bpm, blood pressure was 104/62 mm Hg and respiratory rate was 18 breaths per minute. On exam she was mildly distended with minimal abdominal tenderness. She had a white blood cell count of 14.1 × 10^3^/μl with 85.7% segmented neutrophils, platelets of 344 THOU/μl and hemoglobin of 14.0 g/dl. Metabolic panel was within normal limits (WNL) with creatinine 0.73 mg/dl. Blood glucose was 104 mg/dl. Liver function tests were WNL. Computed tomography (CT) of the abdomen and pelvis with oral and intravenous (IV) contrast was performed, which revealed mild dilation of small bowel and decompressed distal colon with transition point related to ileocolic intussusception with segmental thickening of the cecum and proximal ascending colon, possible cecal mass and trace free fluid (Fig. 1). COVID-19 nasopharyngeal PCR was repeated per hospital policy and was again positive.

She was admitted for initial IV fluid resuscitation and IV ceftriaxone and metronidazole. She had persistent abdominal pain and tenderness and was taken to the operating room the next day for an open bowel resection.

Intraoperatively, the intussusception had spontaneously reduced, and the cecum was notably thickened and edematous without a discrete palpable mass. The terminal ileum was unremarkable. There was an enlarged lymph node within the colonic mesentery of the mid ascending colon. An open right hemicolectomy was performed, and the mesentery was divided at the origin of the right colic artery to include the nodal basin with the specimen.

The patient did well postoperatively. She reported a productive cough and had coarse breath sounds on exam on the second morning after surgery, but she remained on room air with normal oxygen saturations. She had return of bowel function on postoperative Day 1 and was discharged to home on postoperative Day 2 after tolerating a regular diet.

Pathology showed ischemic necrosis, edema, congestion, hemorrhage, transmural acute inflammation, serosal adhesions and acute serositis of the cecum with the impression consistent with intussusception. There was no mass or malignancy identified. The 13 lymph nodes in the specimen were all deemed benign reactive nodes.

## DISCUSSION

SBO remains a common surgical emergency, with approximately one quarter of admissions mandating surgical intervention [5]. In adults, intussusception as the cause of SBO is a rare event, and is typically the result of an identifiable ‘lead point’ including diverticula, polyps, strictures and neoplasia [8]. Approximately half of cases of adult intussusception are secondary to malignancy, mandating surgical resection rather than attempts at endoscopic decompression [8].

Intestinal manifestations of Severe Acute Respiratory Syndrome Coronavirus 2 (SARS-CoV-2) affect 20–70% of infected patients [9]. GI abnormalities including bowel wall thickening, pneumatosis and portal venous gas have been identified in 30% of patients hospitalized with COVID-19 [10]. One study showed that 50% of patients tested positive for SARS-CoV-2 RNA in stool even after negative nasopharyngeal PCR testing and demonstrated viral protein staining in epithelia of the stomach, duodenum and rectum [11]. Taken together, these findings suggest significant involvement of the GI tract in COVID-19 infection.

To our knowledge, we present the first case of intussusception and SBO in an adult with COVID-19 infection, which led to operative intervention. Our patient exhibited no GI symptoms at time of COVID-19 diagnosis, but subsequently developed abdominal pain and GI bleeding secondary to ileocolonic intussusception. Although her abdominal exam was without exquisite tenderness, and fevers and leukocytosis could have been attributed to active COVID-19 infection, her acute presentation, the suspicion of a cecal mass and the typical natural history of colonic lead points in adults causing intussusception led to the decision to not pursue endoscopic decompression or prolonged conservative management, and the patient was taken to the operating room. Intraoperative findings revealed a thickened cecum and mesenteric lymphadenopathy, for which an oncologic right hemicolectomy was performed. Pathology showed necrosis and inflammation of the cecum with benign reactive lymph nodes. Although most common in pediatric cases, her clinical presentation could possibly be attributed to lymphoid hyperplasia secondary to acute viral infection leading to ileocolic intussusception. Such pathophysiology is not completely novel in adults and has been proposed in patients with intussusception and concomitant human immunodeficiency virus (HIV) infection [12, 13].

In hindsight, we may have been able to manage our patient without operative intervention. However in limited reviews the risk of malignancy and sepsis is so high that surgical resection of the affected bowel is still generally recommended in adults [13].

Worldwide, there are four reported cases of SBO thought secondary to COVID-19, all of which were pediatric patients with intussusception [14–17]. All underwent successful nonsurgical reduction initially, but two subsequently required operative intervention. One infant was found to have necrosis of the proximal ileum 9 days after reduction. The patient ultimately died due to multiorgan failure, representing a mortality significantly increased from the typical excellent prognosis of this condition in children [14, 16].

As cases of COVID-19 infection continue across the globe, extrarespiratory manifestations will continue to affect a large proportion of patients. The frequent involvement of the GI tract is typically benign but can potentially present with surgical issues. Although rare, intussusception in COVID-19 positive patients can be a real consequence of the viral infection, and physicians may see such atypical disease processes with increased incidence.
